# The Inclusion of the Microalga *Scenedesmus* sp. in Diets for Rainbow Trout, *Onchorhynchus mykiss*, Juveniles

**DOI:** 10.3390/ani10091656

**Published:** 2020-09-15

**Authors:** Ali Skalli, Joana P. Firmino, Karl B. Andree, Ricardo Salomón, Alicia Estévez, Patricia Puig, Mar Sabater-Martínez, Teresa Hechavarria, Enric Gisbert

**Affiliations:** 1 Laboratory Observatory of the Marchica Lagoon of Nador and Limiting Regions (OLMAN-RL), Multidisciplinary Faculty of Nador, Mohamed 1st University, BP 300, Nador 62700, Morocco; all_skalli@yahoo.es; 2 IRTA, Centre de Sant Carles de la Rápita (IRTA-SCR), Aquaculture Program, Crta. del Poble Nou Km 5.5, 43540 Sant Carles de la Rápita, Spain; joana.firmino@irta.cat (J.P.F.); karl.andree@irta.es (K.B.A.); ricardo.salomon@irta.cat (R.S.); alicia.estevez@irta.cat (A.E.); 3 Andrés Pintaluba S.A., Polígono Industrial Agro-Reus, C. Prudenci Bertrana 5, 43206 Reus, Spain; ppuig@pintaluba.com (P.P.); msabate@pintaluba.com (M.S.-M.); thechavarria@pintaluba.com (T.H.)

**Keywords:** aquafeed, fishmeal replacement, fish oil replacement, microalgae, oxidative stress, fillet quality

## Abstract

**Simple Summary:**

This study aimed to evaluate the suitability of the microalga *Scenedesmus* sp. in diets for rainbow trout juveniles. Considering previous results with similar species, the authors tested the inclusion of this microalga at 5% in diets (48% protein and 18% lipid levels). After 45 days, neither trout growth nor feed efficiency parameters nor fillet proximate composition were negatively affected by the inclusion of the microalga in the diet. In addition, provision of the diet containing the microalga did not lead to observable negative effects on liver or intestinal histological organization and function. Dietary *Scenedesmus* sp. improved the nutritional quality of the fillet in terms of n-3 polyunsaturated fatty acid (PUFA) levels, especially docosahexanoic acid (DHA), although it did alter the color of the fillet. In addition, feeding rainbow trout with diets containing *Scenedesmus* sp. modified the lipid class composition in the liver by increasing the levels of polar phospholipids with regard to triacylglycerides; results that may be attributed to dietary-induced changes in lipid metabolism. Results showed that the green microalga *Scenedesmus* sp. is a safe ingredient for compound feeds in rainbow trout when considering fish growth performance, condition, and health parameters, although the visual appearance of the fillet was affected.

**Abstract:**

A nutritional study was conducted to evaluate the inclusion of the green microalga *Scenedesmus* sp. at 5% (SCE-5) as an alternative fishmeal ingredient. This microalga was tested with four replicates during 45 days using isolipidic (18%), isoproteic (48%), and isoenergetic (1.9 MJ kg^−1^) diets. Fish fed *Scenedesmus* sp. showed similar growth and feed efficiency parameters as the control group. Regarding the digestive function, the SCE-5 diet enhanced the activity of alkaline pancreatic proteases, whereas it did not affect that of intestinal enzymes involved in nutrient absorption. No histological alterations were found in fish fed the SCE-5 diet, although a higher density of goblet cells in the anterior intestine and changes in gut microbiome diversity were found in this group, which collectively suggests positive effects of this green microalga on the intestine. Dietary *Scenedesmus* sp. improved the fillet’s nutritional quality in terms of n-3 polyunsaturated fatty acid (PUFA) levels, although it also increased its yellowish color. The overall results of this study showed that *Scenedesmus* sp. is a safe ingredient for compound feeds in rainbow trout when considering fish growth performance, animal condition, and health parameters, although it substantially affected the color of the fillet that may potentially affect consumers’ preferences.

## 1. Introduction

According to the recently published report from the Food and Agriculture Organization of the United Nations (FAO) entitled “The State of World Fisheries and Aquaculture 2020”, aquaculture will continue to be the driving force behind the growth in global fish production. In particular, aquaculture production is projected to reach 109 million tn in 2030, an increase of 32% (26 million tn) over 2018 [1]. This development heralds a new era of changes led by the aquaculture sector that is supported by an increase in aquafeed production, among other factors [1,2]. Regarding the two conventional ingredients, fishmeal (FM) and fish oil (FO), these raw materials still remain the principal sources of high-quality protein and lipid utilized in feed for carnivorous fish. However, the overexploitation of fisheries resources to produce these ingredients and their growing demand have caused prices of such ingredients to continuously rise. Consequently, the expansion of aquaculture systems based on the use of fishmeal and fish oil as major ingredients for aquafeeds are economically and environmentally unsustainable [2,3,4]. Likewise, according to Duarte et al. [5], there will be an exhaustion of FM and FO by 2040 if oceans are not exploited in a sustainable manner. For this reason, in recent decades the industry and academia have been focused on the search for alternative fish ingredients to use in order to reduce the great dependency on these conventional ingredients. As a result, the aquafeed industry has in recent years introduced substantial changes in diet formulation with the most obvious one being the decrease in the level of ingredients of animal origin coupled with an increase in use of plant-derived ingredients [2,3,4]. Consequently, the search for alternatives to FM and FO for use in dietary formulations has been primarily directed towards protein-rich ingredients and vegetable oils originating from terrestrial plants [4,5,6,7]. However, relatively little attention has been given to the use of novel feed sources, such as macro and microalgae biomass, as alternative raw ingredients in aquafeeds, as reviewed elsewhere [8,9].

Plant protein meals, such as soybean, rapeseed, and corn/wheat gluten meals, have been used successfully in several studies as ingredients for fish feeds [10,11,12,13,14]. Currently, commercial feeds contain blends of plant and fish proteins and oils [2,4]. However, a critical shortcoming of the crop-plant derived protein sources commonly used in aquafeeds is their deficiency in some essential amino acids, such as lysine, methionine, and tryptophan. In addition, plant protein sources contain a wide range of antinutritional factors; hence, high inclusion level of these ingredients can induce negative effects on growth performance [12,13,14,15,16]. As a result, protein sources of plant origin do not represent the ultimate alternative, although their fermentation under controlled conditions may improve their nutritional quality [16]. Thus, the need to find new aquafeed ingredients continues to be a challenging goal for the industry. Any alternative protein source should meet some requirements, such as being easily digestible, contain protein of high-quality, with a sustainable and secure supply, as well as convenient costs. In this regard, owing to their chemical composition, macro- and microalgae appear as promising alternatives aimed at enhancing the nutritive value of conventional feeds, by incorporation into fish diets, as a substitute for fishmeal [8,17,18,19,20,21].

Besides the already well-established applications of microalgae in hatchery systems, where they form the base of the aquatic food chain, they have recently become the subject of great interest as animal feed supplements since they are rich in pigments, such as chlorophylls and carotenoids [22], vitamins [23], antioxidants, and other bioactive compounds, which give them functional properties [24,25]. In addition, microalgae can represent a potential alternative ingredient to supplement the diet of high-value fish species, due to their abundance of high quality proteins reflected by a complete profile of amino acids [8,26,27]. Although there exist several studies evaluating the inclusion of microalgae in diets for marine [18,21,28] and freshwater [29,30,31] species, there still exists limited information about their use in diets for salmonids species, like Atlantic salmon, *Salmo salar* [32,33,34,35], and especially in rainbow trout, *Oncorhynchus mykiss* [34]. Among the extensive number of microalgal species tested so far, the present study is focused on *Scenedesmus* sp., a green microalgae containing 25–35% proteins and rich in essential amino acids, especially lysine [36,37], while it contains relatively low lipid levels [38], with eicosapentaenoic acid (EPA) and docosahexanoic acid (DHA) largely absent among strains [39]. Hence, the objective of the present study was to evaluate the potential inclusion of *Scenedesmus* sp. in a compound diet for rainbow trout juveniles, evaluating its effects on fish performance, fillet quality, and overall fish condition.

## 2. Materials and Methods

All animal experimental procedures were conducted in compliance with the experimental research protocol (reference number 4978-T9900002) approved by the Committee of Ethic and Animal Experimentation of the Institut de Recerca i Tecnologia Agroalimentàries (IRTA) and in accordance with the Guidelines of the European Union Council (86/609/EU) for the use of laboratory animals.

### 2.1. Experimental Diets

Two experimental diets (pellet size = 3.5–4.0 mm) were formulated to have ca. 48% and 18% of protein and lipid levels, respectively, all of them with a high level of inclusion of vegetal ingredients. The inclusion of *Scenedesmus* sp. in diets (5%) was done at the expense of FM, as well as a small fraction of FO in order to obtain an isonitrogenous and isolipidic diet compared to the control one. Thus, the inclusion of *Scenedesmus* sp. at 5% represented 24.4% of the FM replacement. This single dietary inclusion level was chosen based on the results from Vizcaino et al. [18] and Gong et al. [35], who tested the inclusion of *Scenedesmus* sp. in rainbow trout and Atlantic salmon, respectively. In particular, previous results in rainbow trout showed that the inclusion of this green microalgae at 12%, 20%, 25%, and 39% did not result in improvements in somatic growth nor nutrient utilization [18], whereas in Atlantic salmon its dietary inclusion at 10% and 20% did not improve somatic growth and even depressed growth performance at higher levels [35]. The biochemical profile of the tested green microalgae was: 51.1% crude protein (lysine = 23.2 mg g^−1^; methionine = 6.1 mg g^−1^; threonine = 16.9 mg g^−1^; free amino acid content = 15.80 mg/g), 26.3% fat (total saturated fatty acids = 23.4%; total monounsaturated fatty acids = 8.1%; total polyunsaturated fatty acids = 68.4%; EPA + DHA = 0%), 24.1% carbohydrate, 8.4% ash, and 3.4% fiber. Chlorophyll, xanthophyll and carotenoid contents of *Scenedesmus* sp. were 9.98, 8.98, and 0.77 mg/g, respectively (data provided by APSA, Reus, Spain). Diets were manufactured as previously described [39], and their proximate biochemical composition and fatty acid content are shown in Table 1 and Table 2.

### 2.2. Animals, Experimental Conditions, and General Procedures

Rainbow trout (*O. mykiss*) juveniles were purchased from a commercial hatchery (Alevines del Moncayo SA, Vozmediano, Spain), transported by road to the IRTA-Sant Carles de la Ràpita (SCR) facilities (Sant Carles de la Ràpita, Spain), and acclimated for 3 weeks in a 3 m^3^ rectangular fiberglass tank. During this period, fish were fed twice a day (Microbaq 10; Dibaq SA, Fuentepelayo, Spain) at 2% of the stocked biomass. Before the onset of the trial, all fish were individually weighed (BW) and measured for standard length (SL) to the nearest 0.1 g and 1 mm, respectively, and then were distributed into eight 100-L fiberglass cylindrical tanks (25 fish per tank; initial density = 19 kg m^−3^) connected to an IRTAmar^®^ water recirculation system. During the acclimation and experimental periods, water temperature and dissolved oxygen (OXI330; Crison Instruments, Barcelona, Spain) were 17.7 ± 1.7 °C and 8.2 ± 0.8 mg L^−1^, respectively. Water flow rate in experimental tanks was maintained at approximately 9.0 L min^−1^ and water quality was maintained through UV, biological, and mechanical filtration (total ammonia and nitrite were 0 and <0.012 mg L^−1^, respectively); photoperiod was 13 L: 11 D.

Each diet was tested in quadruplicate (tanks) and was offered for juvenile rainbow trout (BWi = 75.5 ± 7 g; SL_i_ = 17.2 ± 0.8 cm; mean ± standard deviation) for a period of 45 days. Feeds were distributed manually four times per day, at the rate of 2.0% of the stocked biomass, which approached apparent satiation. Sampling to monitor fish growth took place at the onset (day 0) and the end of the trial (day 45). For this purpose, all fish were individually measured for BW_f_ and SL_f_ from each tank after being anesthetized with tricaine methanesulfonate (90 mg L^−1^; MS-222; Sigma-Aldrich, Madrid, Spain). At the end of the trial, all fish from each tank were sacrificed with an overdose of anesthetic and their hepatosomatic (HSI) and perivisceral fat (PVI) indexes determined. Fish growth and feed utilization parameters from different experimental groups were evaluated by means of the following indices: Fulton’s condition factor (K) = (BW_f_/SL_f_^3^) × 100; specific growth rate (SGR) in BW (SGR, % per day) = 100 × ((ln BW_f_ − ln BW_i_) × 100)/time (days); feed conversion ratio (FCR, g/g) = FI/(B_f_ − B_i_), where FI was the total feed intake per tank during the experimental period considered (g), and Bi and Bf were the initial and final biomass per tank in grams.

All fish were sacrificed with an overdose of anesthetic, and at least five fish per tank dissected for analytical purposes. In particular, three fish per tank (n = 12 per diet) were used for proximate, fatty acid and lipid class analyses, as well as evaluating the levels of lipid peroxidation and oxidative stress enzymes; five fish per tank were used for evaluating the color of the fillet in both experimental groups (n = 20 per diet), and eight fish per diet (n = 2 per tank) were used to evaluate the potential effects of the inclusion of *Scenedesmus* sp. in the intestinal microbiota and the histological organization of the liver and intestine, as well as for assessing the activity of digestive and oxidative stress enzymes [40]. Finally, five different fish per tank were used for evaluating the impact of diets on hematological and non-specific serological immune parameters.

### 2.3. Liver and Fillet Proximate Composition, Lipid Classes, and Fatty Acid Profiles

Tissues (liver and fillet) were homogenized, and small aliquots were dried (120 °C for 24 h) to estimate their water content. The total fat content from feed and fish tissues was gravimetrically quantified after extraction in a chloroform-methanol solution (2:1) and evaporation of the solvent under a stream of N followed by vacuum desiccation overnight [41]. Protein and carbohydrate contents were determined according to Lowry et al. [42] and Dubois et al. [43], respectively. Ash contents were determined by keeping the sample at 500 to 600 °C for 24 h in a muffle furnace [44]. All chemical analyses were performed in triplicate per fish and feed samples.

In order to evaluate the fatty acid profile of feeds and selected tissues (liver and fillet), methyl esters were extracted twice using isohexane: diethyl ether (1:1, *v*:*v*), purified on thin layer chromatography plates (Silica gel 60, VWR, Lutterworth, UK), and analyzed by gas-liquid chromatography on a Thermo ElectronTraceGC (Winsford, UK) instrument fitted with a BPX70 capillary column (30 m × 0.25 mm id; SGE, UK), using a two-stage thermal gradient from 50 to 150 °C after ramping at 40 °C min^−1^ and holding at 250 °C after ramping at 2 °C min^−1^, helium (1.2 mL min^−1^ constant flow rate) as the carrier gas and on-column injection and flame ionization detection at 250 °C. Peaks of each fatty acid were identified by a comparison with known standards (Supelco Inc., Madrid, Spain) and a well characterized fish oil, and they were quantified by means of the response factor to the internal standard, 21:0 fatty acid, added prior to transmethylation, using a Chrom-card for Windows (TraceGC, Thermo Finnigan, Monza, Italy). Results of fatty acid content are expressed as a percentage of total fatty acids (TFA).

Lipid class analyses were only performed in liver samples by means of high-performance thin-layer chromatography [45]. Approximately, 10 µg of lipids were applied as a 2 mm streak and the plate developed to two-thirds distance with a mixture of methyl acetate/isopropanol/chloroform/methanol/0.25% aqueous KCl (25:25:25:10:9 *v*:*v*) to separate polar lipid classes, and then fully developed with isohexane/diethyl ether/acetic acid (85:15:1 *v*:*v*). After separation, bands were identified by charring the plates at 100 °C for 30 min after spraying with 3% (*w*/*v*) aqueous cupric acetate containing 8% (*v*/*v*) phosphoric acid and quantified by scanning densitometry using a GS 800 Calibrated Densitometer (Bio-Rad, Bio-Rad Laboratories, Inc, Hercules, CA, USA). The identities of individual lipid classes were confirmed by a comparison with standards.

### 2.4. Analysis of Fillet Color

Fillet color was instrumentally assessed by using a Chroma meter (Minolta^®^ Camera Co., Ltd., CR-300, Osaka, Japan), which compares the reflectance of light from an object (fish fillet) with that of a standard calibration plate. Lightness (L*, negative for blackness and positive for whiteness), red–green chromaticity (a*, negative for greenness and positive for redness), and yellow–blue chromaticity (b*, negative for blueness and positive for yellowness) were measured for each fillet. Five fish per tank (right-side fillet of each fish) were used for the instrumental colorimetric analysis. The colorimetric values of the white muscle in three places (two dorsal, one ventral) in a single fillet were analyzed, and the average value was used for calculations. Values were obtained from the same area of the fillet for all fish analyzed. Values for a* and b*, representing redness and yellowness respectively, were used to calculate the hue angle (h*) and the level of color saturation (C*).

### 2.5. Levels of Lipid Peroxidation and Activity of Oxidative Stress Enzymes

Quantification of lipid peroxidation [LPO, nmol malondialdehyde (MDA) 100 g^−1^] in the fillet and liver was conducted using the thiobarbituric acid reactive substances method described by Solé et al. [46]. Homogenized samples, prepared for the determination of LPO, were also used to measure the activity of antioxidant stress enzymes. Superoxide dismutase (SOD) was measured using a commercial kit (catalogue number 19160) from Sigma-Aldrich (Madrid, Spain). Catalase (CAT) activity was measured by the decrease in absorbance at λ = 240 nm (e = 40 M^−1^ cm^−1^) using 50 mM H_2_ O_2_ as substrate [47]. Glutathione S-transferase (GST) was assayed by the formation of glutathione chlorodinitrobenzene adduct at λ = 340 nm (e = 9.6 mM^−1^ cm^−1^), using 1 mM 1-chloro-2,4-dinitrobenzene and 1 mM glutathione as substrates [48]. Glutathione reductase (GR) activity was determined by measuring the oxidation of NADPH at λ = 340 nm (e = 6.22 mM^−1^ cm^−1^), using 20 mM glutathione disulphide and 2 mM NADPH as substrates [49]. Total glutathione peroxidase (GPX) was determined by measuring the consumption of NADPH at λ = 340 nm (e = 6.22 mM^−1^ cm^−1^), using 75 mM glutathione and 8.75 mM NADPH as substrates [50]. Enzyme activities were expressed as specific enzyme activities (mmol min^−1^ mg protein^−1^), and soluble protein was determined by the Bradford method [51]. All assays were conducted in triplicate at 25 °C and absorbance read using a spectrophotometer (Tecan^TM^ Infinite M200; Techan Group Ltd., Männedorf, Switzerland).

### 2.6. Gut Microbiota

Potential changes to the intestinal microbiota by means of the polymerase chain reaction-restriction fragment length polymorphisms (PCR-RFLP) were performed as described by Gómez-Conde et al. [52]. This approach was chosen as a proxy for evaluating the impact of the inclusion of the microalga *Scenedesmus* sp. in the intestinal microbiota for its simplicity and cost effectiveness. Intestinal contents from 4 fish per tank were used for the analysis; two fish per replicate and two replicates per tank. The entire intestine was collected and fixed in ethanol at the time of sacrifice, and later were opened longitudinally with sterile scissors and the mucosal layer scraped from the intestinal walls using a sterile stainless steel spatula. Individual samples of ~0.4 g from the intestinal mucosa were processed for total DNA extraction (QIAmp DNA Stool Mini Kit; Qiagen Inc., Chatsworth, CA, USA) according to the kit manufacturer’s instructions. The purified DNA was maintained at −20 °C until use. The primers 5′-CTACGGGAGGCAGCAGT-3′ and 5′-CCGTCWATTCMTTTGAGTTT-3′, corresponding to regions I and II of the 16 S rRNA gene, were used to amplify a 500- to 600-bp product (size varying according to taxa). Amplifications were performed in a final volume of 50 μL using a PCR-Master Mix containing 1.25 IU of Taq polymerase (Applied Biosystems, Foster City, CA, USA), 100 ng of DNA template, 2 mM MgCl_2_, 0.2 µM of each primer, and utilized the following cycling conditions: 94 °C for 5 min, followed by 35 cycles of 94 °C for 1 min, 45 °C for 1 min (with an increase of 0.1 °C each cycle), and 72 °C for 1 min and 15 s. The last extension cycle was continued for 5 min. Aliquots of 6 µL of the amplified DNA fragments were digested in separate tubes with restriction endonucleases (Alu I, Rsa I, Hpa II, Sau 3AI, or Hha I; New England Biolabs, Ipswich, MA, USA) in a total volume of 12 µL. The entirety of each endonuclease digestion was loaded into wells and resolved in 2% agarose gels run for 60 min at 150 V. The DNA bands were visualized using a digital imaging system (GeneFlash, SynGene, Cambridge, UK), and the resulting band patterns were analyzed for cladistics based on size and number of bands to produce the dendograms shown (GeneTools; SynGene, Frederick, MD, USA).

### 2.7. Hematological and Serological Immune Parameters

Blood (1.5 mL) was taken from anesthetized fish by caudal puncture with lithium-heparinized syringes and centrifuged (2000× *g* for 20 min at 4 °C) to separate serum, aliquoted and frozen at −80 °C until their analysis. Hematocrit (Hct, %), was determined in fresh blood according to Blaxhall and Daisley [53]. Serum protease activity was quantified using the azocasein hydrolysis assay [54]. The lysozyme activity in serum was measured according to the method of Ellis [55] and each unit (KU mL^−1^) is defined as the amount of sample causing a decrease in absorbance of 0.001 per min. The assay for alternative complement pathway (ACP) was determined following the technique described by Sunyer et al. [56] with minor modifications for ELISA plates; the results (ACH_50_ mL^−1^) were expressed in alternative complement units per mL, which are defined as the titer at which 50% hemolysis is produced. For the bacteriolytic test, bacteria (*Escherichia coli*) were grown for 20 h in 20 mL of lysogenic broth at 37 °C in an orbital incubator at 200 rpm. A 1:100 bacterial suspension was chosen to give an optical reading of 0.5 to 0.6 at a wavelength of λ = 540 nm when added to the serum dilution (1:1 bacterial suspension: serum dilution) and blank with sterile Luria-Bertani medium. The mixture was placed for 1 h at 37 °C on an orbital incubator (200 rpm). To study the bactericidal kinetics of fish serum, a 0.5-mL aliquot was withdrawn at intervals of 30 min and read at λ = 540 nm with a microplate reader (Tecan Infinite M200; Tecan Group Ltd., Barcelona, Spain). Results are given as fold increase of the absorbance. Serum peroxidase (catalogue number MAK092) and myeloperoxidase (MPO, catalogue number MAK069) activities (µU mL^−1^) were measured using a commercial kit from Sigma-Aldrich (Madrid, Spain). In addition to the role of myeloperoxidase as an essential part of the anti-microbial system, we also considered its activity in the serum as an inflammatory marker [57].

### 2.8. Organization and Functionality of the Digestive System

For assessing the impact of the dietary inclusion of *Scenedesmus* sp. on the digestive system organization and functionality, sacrificed fish were dissected on a glass plate at 0–4 °C. Stomach and pyloric caeca were sampled for measuring the activity of gastric (pepsin) and pancreatic proteases (trypsin, chymotrypsin, and total alkaline protease activities), bile salt-activated lipase, and α-amylase, whereas the anterior and posterior regions of the intestine were obtained for measuring the activity of several brush border enzymes (alkaline phosphatase, maltase, and aminopeptidase N). Enzyme extracts were prepared and spectrophotometric analyses performed as recommended by Solovyev and Gisbert [58] in order to prevent sample deterioration. Pyloric caeca samples were homogenized in 5 volumes (wet weight; *ww/v*) of distilled water at 4 °C for 1 min, followed by a sonication process of 30 s [59]. Intestinal samples were homogenized in 30 volumes (*w/v*) of ice-cold mannitol (50 mM), Tris-HCl buffer (2 mM) pH 7.0 as described in Gisbert et al. [60].

Total alkaline protease activity was measured using azocasein (0.5%) as substrate in Tris-HCl 50 nmol L^−1^ (pH = 9). One unit (U) of activity was defined as the nmoles of azo dye released per minute and per mL of tissue homogenate (λ = 366 nm) [61]. Trypsin activity was assayed using BAPNA (N-α-benzoyl-DL-arginine p-nitroanilide) as substrate; one unit of trypsin per mL (U) was defined as 1 μmol BAPNA hydrolyzed min^−1^ mL^−1^ of enzyme extract (λ = 407 nm) [62]. Chymotrypsin activity was quantified using BTEE (benzoyl tyrosine ethyl ester) as substrate, and its activity (U) corresponded to the μmol of substrate hydrolyzed min^−1^ mL^−1^ of enzyme extract (λ = 256 nm) [63]. Alpha-amylase activity was determined using 0.3% soluble starch as substrate, and its activity (U) was defined as the amount of starch (mg) hydrolyzed during 30 min per mL of homogenate (λ = 580 nm) [64]. Bile salt-activated lipase activity was assayed for 30 min using p-nitrophenyl myristate as substrate; and its activity (U) was defined as the amount (nmol) of substrate hydrolyzed per min per mL of enzyme extract (λ = 405 nm) [65]. Pepsin was quantified using 2% hemoglobin as substrate in 1 N HCl buffer as substrate, and its activity (U) defined as the nmol of tyrosine liberated per min per mL of tissue homogenate (λ = 280 nm) [66]. Alkaline phosphatase was quantified using 4-nitrophenyl phosphate (PNPP) as substrate. One unit (U) was defined as 1 μmol of p-nitrophenol (pNP) released min^−1^ mL^−1^ of brush border homogenate (λ = 407 nm) [60]. Aminopeptidase-N was determined using 80 mM sodium phosphate buffer (pH = 7.0) and L-leucine p-nitroanilide as substrate [67], and one unit of enzyme activity (U) was defined as 1 μg nitroanilide released per min per mL of brush border homogenate (λ = 410 nm). Maltase activity was determined using d (+)-maltose as substrate in 100 mM sodium maleate buffer (pH = 6.0) [68]. One unit of maltase (U) was defined as μmol of glucose liberated per min per mL (λ = 420 nm). Soluble protein of crude enzyme extracts was quantified by means of the Bradford’s method [51] using bovine serum albumin as standard. All enzymatic activities were measured at 25–26 °C and expressed as specific activity defined as units per mg of protein (U mg protein^−1^). All the assays were made in triplicate (methodological replicates) for each tank and the absorbance was read using a spectrophotometer (Tecan^TM^ Infinite M200, Männedorf Switzerland).

For histological purposes, formalin-fixed liver and anterior and posterior intestine samples were dehydrated in a graded series of ethanol, cleared with xylene, embedded in paraffin and cut in serial sections (3–5 μm thickness). Transverse sections were stained with hematoxylin–eosin, observed with a light microscope (Leica DM LB; Leica Microsystems, Wetzlar, Germany) and photographed (Olympus DP70 Digital Camera; Olympus Imaging Europa GmbH). Digital images (600 dpi) were processed and analyzed using an image analysis software package (ANALYSIS; Soft Imaging Systems GmbH; Münster Germany). Measurements of total goblet cell number (full and empty) and villi height were based on the analysis of 8–10 randomly chosen fields from each cut [40].

### 2.9. Statistical Analyses

Data are presented as mean ± standard error (SE) calculated from mean values obtained from each replicate tank (n = 4). Data expressed as percentage were arcsine square root transformed before analyzed. Data were compared by means of a *t*-test. In addition, the levels of lipid peroxidation and activity of oxidative stress enzymes were also analyzed by an one-way ANOVA in order to evaluate differences between the fillet and the liver (data normally distributed, Kolmogorov–Smirnov test and homogeneity of variances previously tested).

## 3. Results

### 3.1. Growth, Condition Factor, and Somatic Indexes

The inclusion of *Scenedesmus* sp. at 5% in compound diets for rainbow trout did not affect the growth in terms of BW and SL, nor their condition factor of juveniles (*t*-test; *p* > 0.05; Table 3). In addition, no statistically significant differences were found in HSI and PVI values between both experimental groups (ANOVA, *p* > 0.05; Table 3).

### 3.2. Fillet Quality: Color and Biochemical Composition

Fillet lightness (L*) and redness (a*) color values were not affected by the inclusion of *Scenedesmus* sp. in diets for rainbow trout (Table 4; *t*-test, *p* > 0.05). However, yellowness (b*) color values were significantly affected by the diet, being 3.8 times higher in the fillet of the SCE-5 group in comparison to the CTRL group (*t*-test, *p* < 0.05). Changes in b* also resulted in significant differences in C* and h* parameters between both groups (*t*-test, *p* < 0.05). An example of fillet and serum color showing their yellowness may be found in the Figure S1.

The proximate biochemical composition of the fillet was not affected by the inclusion of *Scenedesmus* sp. in the experimental diet (Table 5; *t*-test, *p* > 0.05). Regarding the fatty acid profile, no differences in saturated fatty acids and total n-6 polyunsaturated fatty acids (PUFA) were found among dietary groups (Table 6; *t*-test, *p* > 0.05). However, statistically significant differences were found in terms of total monounsaturated fatty acids in the fillet of fish from the CTRL group that were higher than in fish fed the SCE-5 diet (*t*-test, *p* < 0.05). In addition, levels of docosapentanoic acid (DPA, 22:5 n-3), docosahexanoic acid (DHA, 22:6n-3) and total n-3 PUFA were higher in the fillet from the SCE-5 group in comparison to their congeners fed the CTRL diet, whereas their levels in α-linolenic acid (ALA; 18:3-n-3) decreased (*t*-test, *p* < 0.05).

### 3.3. Liver Biochemical Composition

No differences in protein, carbohydrates and ash contents were found in the liver of rainbow trout juveniles fed the SCE-5 diet in comparison to the control group (Table 5; *t*-test, *p* > 0.05), whereas lipid content significantly decreased in the livers of fish fed the SCE-5 diet (*t*-test, *p* < 0.05). Regardless of changes in lipid content, no differences in the fatty acid profile in livers from both dietary groups were found (Table 6; *t*-test *p* > 0.05).

In contrast, the dietary inclusion of *Scenedesmus* sp. changed the lipid class composition in the liver of rainbow trout juveniles (Table 7; *t*-test, *p* < 0.05). In particular, the content in total phospholipids in the liver of fish fed the SCE-5 diet increased in comparison to the control group. This increase was due to an increment in the levels of phosphatidylcholine, phosphatidylserine, phosphatidylinositol and lysophosphatidylethanolamine (*t*-test, *p* < 0.05). Higher total phospholipid levels in the liver of fish fed the SCE-5 diet resulted in lower total neutral lipid contents; in particular, levels of cholesterol and free fatty acids were lower in the liver of rainbow trout fed the SEC-5 diet as compared to the control group (*t*-test, *p* < 0.05).

### 3.4. Lipid Peroxidation Levels and Activity of Oxidative Stress Enzymes

Similar LPO values were observed in the fillet and liver of rainbow trout fed different experimental diets (Table 8; *t*-test, *p* > 0.05). Higher SOD activities were found in the liver in comparison to those measured in the muscle (ANOVA, *p* < 0.05), even though no statistically significant differences were found between dietary groups irrespectively to the tissue considered (*t*-test, *p* > 0.05). In contrast, higher activity values in CAT, GST, GR, and GPX were found in the fillet of fish fed different diets when compared to the liver (ANOVA, *p* < 0.05), regardless of the diet considered.

### 3.5. Organization and Functionality of the Digestive System

The activity of total alkaline proteases, trypsin, and chymotrypsin were significantly affected by the inclusion of *Scenedesmus* sp. in the diet (Table 9; *t*-test, *p* < 0.05). In particular, rainbow trout juveniles fed the SCE-5 diet showed higher specific activity values than those fish fed the CTRL diet. In contrast, no statistically significant differences were found with regard to the other pancreatic digestive enzymes analyzed, α-amylase, and bile salt-activated lipase (*t*-test, *p* > 0.05). No statistically significant differences in the activity of pepsin and the three assayed intestinal brush border enzymes were detected in rainbow trout juveniles fed the SCE-5 diet compared to the CTRL group (*t*-test, *p* > 0.05).

The histological organization of the liver and anterior and posterior regions of the intestine were normal, and no histopathological alterations in the hepatic parenchyma nor intestinal mucosae were observed due to the inclusion of *Scenedesmus* sp. in the diet for rainbow trout. In both experimental groups, lipid accumulation in the liver was moderate, whereas no signs of steatosis were found, and the presence of melanomacrophage centers was rare (Figure S2). No changes in villi length in the anterior and posterior intestinal regions were found between dietary groups (Table 10; *t*-test, *p* > 0.05). In contrast, goblet cell density increased in the anterior intestine of rainbow trout juveniles fed the SCE-5 diet in comparison to their congeners fed the CTRL diet (*t*-test, *p* < 0.05), whereas no differences in this parameter were found in the posterior intestine (*t*-test, *p* > 0.05).

### 3.6. Hematological and Non-Specific Serological Immune Parameters

The inclusion of *Scenedesmus* sp. in the diet for rainbow trout did not significantly affect the hematocrit values between experimental groups, nor the non-specific immune parameters and peroxidase and MPO levels measured in the serum of animals (Table 11; *t*-test, *p* > 0.05).

### 3.7. Microbiota Analysis

**Figure 1:**
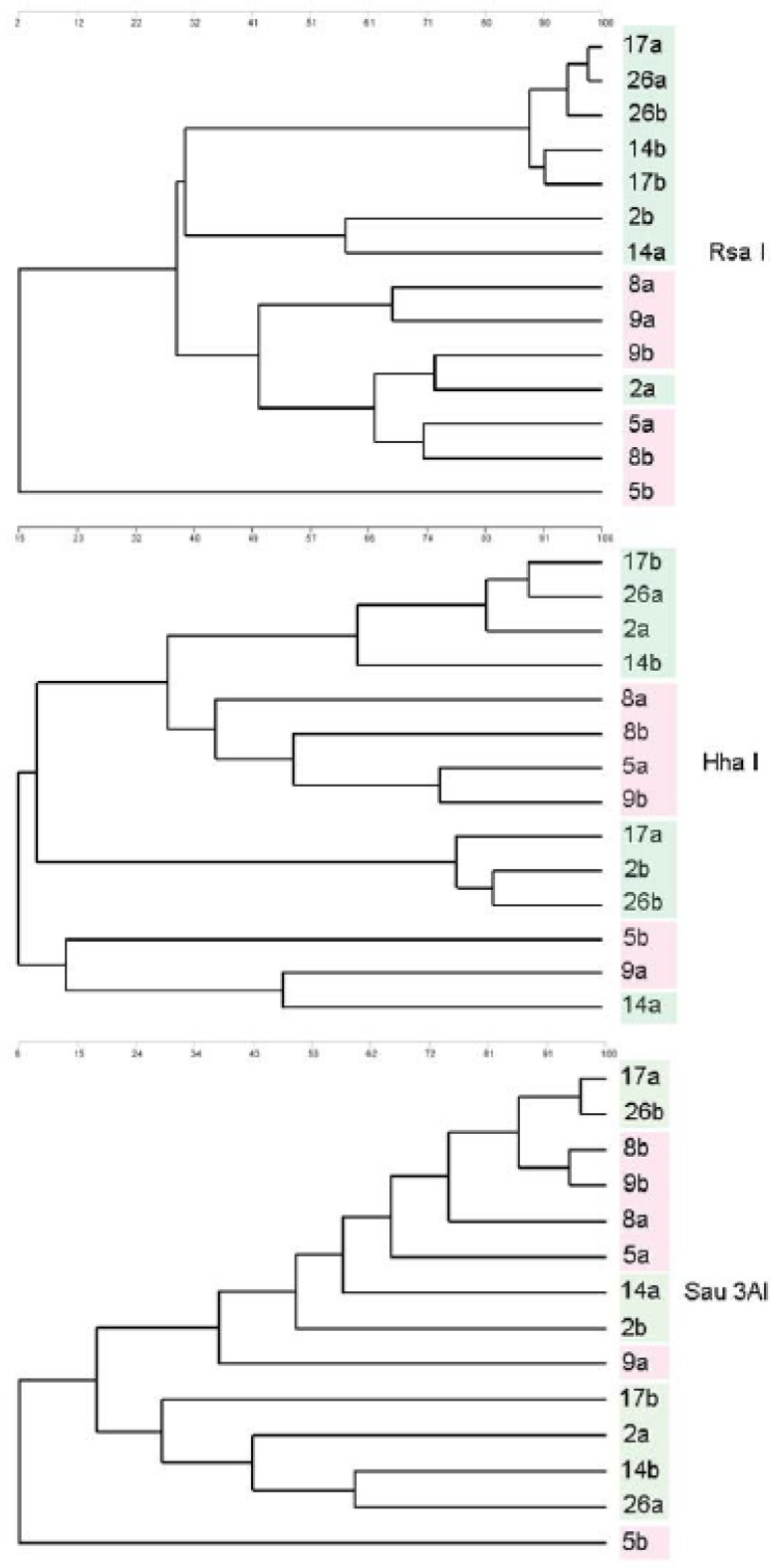
Dendograms resulting from the cluster analysis of the RFLP patterns obtained from the gut microbiome of rainbow trout (*O. mykiss*) using different restriction enzymes (HhaI, RsaI, and Sau3AI). Fish of each dietary group (CTRL diet = red color, and SCE-5 diet = green color) are compared. Different numbers correspond to different analyzed samples, each containing two fish. Replicates are denoted by either “a” or “b”.

As fish were fasted for ca. 24 h prior to sampling, their gut was mostly empty and therefore, bacteria present in the samples were from the intestinal mucosa (autochthonous microbiota) rather than from the chime (allochthonous microbiota). Restriction enzymes used in the PCR-RFLP analysis provided differing results depending on the frequency of cutting, which is dependent on the sequences obtained by PCR amplification. The three enzymes *Hha I*, *Rsa I*, and *Sau 3AI* provided clear restriction digestion patterns, while the cladistic analysis showed clustering of fish fed the SCE-5 diet apart from those fed the CTRL diet (Figure 1).

## 4. Discussion

One of the main obstacles in the development of a sustainable aquaculture industry is its direct dependency on the more expensive conventional ingredients of animal origin (i.e., fishmeal and fish oil) that are the principal sources of high-quality protein and lipid utilized to feed carnivorous fish species [2,4]. Therefore, it is widely recognized that one of the greatest challenges for any future increases in productivity in the aquaculture sector is to identify, test, and validate high quality sources of alternative proteins and oils for aquafeeds. Among different alternative ingredients, microalgae have shown promise as potential replacements for FM and FO in feeds for salmonids and other finfish because of their elevated fatty acid profiles and high protein content, depending on the species considered [8]. Various species of microalgae, including *Spirulina*, *Nannochloropsis*, *Chlorella*, *Isochrysis*, *Tetraselmis*, *Secenedesmus*, and *Schizochytrium* spp. have proven to be viable protein and lipid sources in aquafeeds [8,69,70]. Under this context, the authors decided to test the suitably of *Scenedesmus* sp. in diets for juveniles of rainbow trout considering the promising results obtained in gilthead seabream (*Sparus aurata*) [18], Atlantic salmon [35], and several freshwater species [28], including rainbow trout [71].

Under present experimental conditions, the inclusion of *Scenedesmus* sp. at 5%, representing a 24.4% of FM replacement, did not compromise somatic growth in rainbow trout, as fish fed the SCE-5 diet showed similar growth performance in terms of BW, SL, SGR, and body condition factor, as well as similar values in terms of FCR and FI. These results were in agreement with those found in gilthead seabream fed diets containing graded levels of *Scenedesmus almeriensis*, where the replacement of FM from 12 to 39% by this microalga did not compromise growth performance in this marine species [18]. However, there exist other studies in which the FM substitution by different microalgal species impaired fish performance. For instance, in diets for rainbow trout, *S. almeriensis* produced in agro-industrial wastewater evaluated at 1.3 to 10% of inclusion decreased somatic growth, results that were attributed to the lower digestibility of the microalga protein [70]. In contrast, other authors have recently reported that regardless of showing good values for digestibility, the inclusion in diets of a blend of several microalgae, such as *Nannochloroposis* sp. (7%) and *Isochrysis* sp. (2.4%), *Nannochloroposis* sp. (7%) and *Schyzochitrium* sp. (2.5%), and *Nannochloropsis* sp. (7%) and *Isochrysis* sp. (2.4%), impaired growth performance in rainbow trout. The former results were associated to a reduction in feed intake due to changes in diet palatability [71], results that also contributed to poorer FCR values. These findings were similar to those found in Atlantic cod (*Gadus morhua*) fed a combination of *Nannochloropsis* and *Isochrysis* spp. replacing 15 and 30% of FM [19]. Although comparing feed efficiency parameters among different studies may lack in veracity due to differences in feed formulation, feeding practices and rearing conditions, FCR values recorded in the present trial were within the range of values found in other studies dealing with rainbow trout [71,72,73]. Under present experimental conditions, neither FCR nor FI were affected by the inclusion of *Scenedesmus* sp. in the feed for rainbow trout, similarly to Vizcaíno et al. [18], whereas other studies have reported an increment in FCR values with increasing inclusion rates of this microalga in rainbow trout [70] and Atlantic salmon [35]. Such reduced FCR and FCI values have been associated to lower bioavailability of nutrients [35], whereas others have correlated it to changes in diet palatability [70].

The replacement of FM by *Scenedesmus* sp. did not vary the proximate composition nor oxidative stress parameters of the fillet, whereas it affected its fatty acid profile. In particular, the SCE-5 diet reduced the amount of mono-unsaturated fatty acids (18:1n-9, 20:1n-9) and increased total n-3 PUFA levels in the fillet of rainbow trout. It is generally accepted that the fatty acid profiles of the fillet and liver are closely similar to the fatty acid content of the diet [74]. Thus, changes in the fillet of levels of oleic (18:1n-9) and eicosenoic (20:1n-9) acids might be correlated to the lipid content of *Scenedesmus* sp., since a small fraction of FO was replaced in the SCE-5 diet by the microalga (7.6% of FO replacement) in order to formulate isolipidic diets. These findings were not found in Atlantic salmon fed 10–20% of *Scenedesmus* sp. [35], differences that may be attributed to different basal feed composition between both studies. The higher content of total n-3 PUFA, especially docosahexanoic acid and DHA, in the fillet with regard to their content in experimental diets showed their selective retention. Relative resistance of the DHA to β-oxidation and high specificity of fatty acyl transferases for this n−3 LC-PUFA may be possible mechanisms for its selective deposition in tissues [74,75]. In addition, the fillet of rainbow trout fed the SCE-5 diets showed higher levels of DHA, DPA, and total n-3 PUFA levels when compared to the control group. Considering the absence of EPA and DHA in in the evaluated *Scenedesmus* sp., their fillet content may be attributed to their endogenous biosynthesis from their precursor, ALA [76,77]. In Atlantic salmon, total n-3 PUFA levels of the fillet increased when *Scenedesmus* sp. was included at 10%, whereas a higher level of dietary inclusion (20%) did not modify them. These changes in the fillets of Atlantic salmon were attributed to changes in ALA, as well as to modest changes in eicosapentaenoic acid (EPA, 20:5n-3) and DHA [35]. In our study, no changes in total n-6 PUFA content of the fillet were found between dietary groups, whereas such an increase in these groups of fatty acids was found in Atlantic salmon fed diets containing 10 and 20% of *Scencedesmus* sp.; changes that were mainly attributed to the higher content of linoleic acid (LA, 18:2n-6) in salmon feed. These results from rainbow trout are of special relevance regarding the incorporation of low levels of *Scenedesmus* sp. in diets, since fish are recognized as one of the most important sources in human nutrition for fatty acid content.

Regarding fillet quality in terms of its color, present results were in agreement with those reported using *S. almeriensis* produced in agro-industrial wastewater [70]. In both studies, the dietary inclusion of *Scenedesmus* sp. resulted in an increase in the yellow coloration (b*) of the fillet, although the level of magnitude of this change varied depending on the study. In particular, b* values in our study incorporating *Scenedesmus* sp. at 5% were double those measured when *S. almeriensis* was included at 10% in diets for rainbow trout [70]. The increase in yellow fillet coloration may be attributed to the presence of carotenoids, especially lutein, in this green microalga. Microalgae belonging to the genus *Scenedesmus* sp. are reputed for their high content of lutein that may reach up to 0.5% depending on production conditions [78]. Thus, differences between the yellow color between both studies may be due to differences in carotenoid contents between the tested *Scenedesmus* spp. Although FM replacement by *S. almeriensis* did not affect the quality properties of the fillet as a sensorial test panel indicated [70], the increase in the yellow color of the fillet may substantially affect the consumers’ acceptance. This may potentially result in an attitude of rejection towards such a yellowish rainbow trout fillet, although this issue may be corrected by the use of finishing diets at later stages of the production cycle [79,80]. In addition, how this yellowish fillet coloration may be affected when rainbow trout fillets are colored with natural or synthetic carotenoid pigments should also be further assessed. However, it should not be excluded that such a yellow fillet coloration added to its good nutritional profile in terms of n-3 HUFA contents, may be viewed positively (i.e*.,* “golden” fillet trout) as a new product in an increasingly diversified gourmet seafood market. Nevertheless, this is just a hypothesis that needs to be further evaluated by a taste panel of consumers, as well as marketing specialists of the seafood industry.

The inclusion of *Scenedesmus* sp. improved the digestive condition of rainbow trout juveniles. In particular, regarding to pancreatic enzymes, fish fed the SCE-5 diet showed higher specific activities in total alkaline proteases, trypsin, and chymotrypsin, whereas activity of carbohydrases (α-amylase) and lipases (bile salt-activated lipase) were not affected. Pancreatic digestive enzymes are widely considered as indicators of the digestive capacity in fish, whereas brush border intestinal enzymes are biomarkers of intestinal absorptive capacities and enterocytes’ integrity. Under current experimental conditions, the inclusion of *Scenedesmus* sp. in the rainbow trout diet did not compromise the digestive function as confirmed by results from pancreatic, gastric and intestinal enzymes analyses. In particular, the increase in the secretion of pancreatic proteases may not be attributed to a reduction in plant protein sources in experimental feeds [12], since these ingredients did not vary between CTRL and SCE-5 diets. Thus, such an increase in alkaline pancreatic proteases may be either attributed to a need for a higher proteolytic digestive capacity for properly digesting the rigid and extremely resistant cell wall of *Scenedesmus* sp. [81] and/or such results might be linked to a higher soluble protein content of the SCE-5 diet that promoted protease secretion [82] by means of gastro-intestinal hormones [83]. However, such effects were not observed with regard to pepsin, an acid protease; results that might be attributed to differences in their dietary regulation [84]. Regarding the intestinal function, functional data retrieved from the assessment of brush border enzymes coupled with histological data, revealed that the inclusion of *Scenedesmus* sp. in the diet for rainbow trout juveniles did not damage the intestinal mucosa or impair the absorption of nutrients. These results were in agreement with those already reported in gilthead seabream fed diets containing graded levels of *S. almeriensis* [18]. These results indicated that *Scenedesmus* sp. was safe in terms of intestinal health and did not result in enteritis in rainbow trout. Furthermore, these findings were also supported by the absence of differences in terms of growth performance and feed efficiency parameters, as already discussed. Although the histological organization of the intestinal mucosa was similar between the two dietary groups, we found an increase in the density of goblet cells in the anterior intestine of rainbow trout fed the SCE-5 diet. The intestine is a key player in food digestion and nutrient absorption, providing a physiologic and immunologic barrier to a wide range of microorganisms and foreign substances. Therefore, an increase in the density of goblet cells would benefit the host by providing an effective immune barrier against potentially pathogenic gut bacteria [85]. Mucus produced by intestinal goblet cells can contain lysozymes, immunoglobins, lectins, and antibacterial peptides that counter pathogens and toxins, as well as playing a key role in the establishment of the commensal intestinal microbiota [52,86]. Consequently, the increase in goblet cell density in the anterior intestine can be interpreted as an enhancement of the intestinal innate immune function in fish resulting from being fed *Scenedesmus* sp. In addition, FM replacement by *Scenedesmus* sp. did modify the gut microbiota composition, showing a more homogeneous gut bacterial community as suggested by results from the PCR-RFLP analysis. The chosen methodological approach did not allow a detailed analysis of changes in microbiome composition and abundance between both experimental groups, but it served as a proxy of the effect of the diet on the gut microbiome. In a similar study in rainbow trout fed an experimental diet containing 5% *Schyzochytrium* sp., authors found that the increased microbial diversity could be indicative of the microbial community responding to the availability of a different dietary ingredient, such as an additional fermentable substrate in the form of the whole-cell microalgae supplement [85]. Similar results were found in Nile tilapia (*Oreochromis niloticus*) fed a diet supplemented with 1.2% of *Schyzochytrium* sp. [87]. In both former studies, dietary changes on microbiome diversity did not result in changes on the microbial community structure. In this sense, a more diverse microbiome in rainbow trout fed the SCE-5 diet could therefore represent a reflection of the need for additional plasticity in the structure of their microbiome, in order to aid the digestion and assimilation of the microalgal meal included in their diet.

Regarding the liver, the inclusion of *Scenedesmus* sp. in the rainbow trout diet did not alter the histomorphological organization of the hepatic parenchyma, as no substantial changes in liver fat deposits were observed within hepatocytes; results that were in agreement with hepatic oxidative stress parameters in fish fed the SCE-5 diet. In addition, no hepatic damage was observed in rainbow trout juveniles fed the SCE-5 diet as similar MPO levels were found between both dietary groups [88]. Although no differences in the fatty acid profile in the liver were found between both experimental groups, the SCE-5 diet changed the lipid class composition in this accessory digestive gland. The replacement of FM and FO by *Scenedesmus* sp. increased the proportion of phospholipids, a change that was also associated to a reduction in cholesterol and free fatty acids in the liver. These modifications indicated dietary-induced changes in lipid metabolism without being affected by oxidative stress. Thus, the lower neutral lipid levels found in the liver of fish fed the SCE-5 diet might be attributed to a reduction in hepatic triacylglycerol synthesis and stimulation of hepatic peroxisomal β-oxidation [89]. In addition, considering that the dietary inclusion of *Scenedesmus* sp. amounts to 7.6% of FO replacement as compared to the control diet, this may explain the higher liver cholesterol content in rainbow trout fed the CTRL diet due to the higher squalene present in FO that is a precursor of this kind of neutral lipid. These results indicated that the liver of rainbow trout fed the SCE-5 diet might be in a better health condition due to its higher content in phospholipids, especially phosphatydylcholine (PC), phosphatidylserine (PS), phosphatidylinositol (Pi), and lysophosphatidylethanolamine (LysoPE), suggesting a lowered propensity to develop hepatic steatosis [90,91].

Dietary *Scenedesmus* sp. did not impair nor enhance the humoral non-specific immune response in rainbow trout. Although other studies have reported that the inclusion of different microalgal species in aquafeeds regulated innate and adaptive immunity [92,93,94]. There exist few studies evaluating the immunomodulating properties of *Scenedesmus* sp. In particular, *S. ovalternus* supplement (4%) enhanced the resistance of gibel carp (*Carassius gibelio*) against *Aeromonas hydrophila* after overwintering, whereas immune markers were observed to be unaffected or even decrease before the bacterial challenge [95]. Additionally, *Scenedesmus* sp. was also reported to promote immunity in broiler chickens [96]. Differences among studies with similar or different microalgal species may be due to variations in their inclusion levels, diet formulation, microalgal cell wall composition and content in bioactive compounds [97]. Nevertheless, in order to access the real immune potential of the dietary inclusion of *Scenedesmus* sp., further analysis considering an in vitro or in vivo infectious challenge should be considered.

## 5. Conclusions

The inclusion of the green microalga *Scenedesmus* sp. at 5% in a compound diet (48% protein, 18% lipid) for juveniles of rainbow trout, which resulted in 24.4 and 7.6% of FM and FO replacement, respectively, did not negatively affect growth performance and feed efficiency parameters. Regarding the digestive function, the dietary inclusion of the microalga enhanced the activity of alkaline proteases secreted by the pancreas, whereas it did not affect the activity of intestinal brush border enzymes involved in nutrient absorption. In addition, no histological alterations were found in fish fed the SCE-5 diet; this dietary group did show a higher density of goblet cells in the anterior intestine, as well as changes in gut microbiome diversity, which indicated probable positive effects of this green microalga on the intestine. The liver was also positively impacted by dietary *Scenedesmus* sp., especially in terms of polar lipid content that were increased with regard to triacylglycerides, results that may be attributed to dietary-induced changes in lipid metabolism. Dietary *Scenedesmus* sp. improved the nutritional quality of the fillet in terms of n-3 PUFA levels, especially DHA, although it increased its yellowish color. The overall results of this study showed that the green microalga *Scenedesmus* sp. is a safe ingredient for compound feeds in rainbow trout when considering fish growth performance, animal condition, and health parameters, although it substantially affected the visual appearance of the fillet that may potentially affect consumers’ preferences.

## Figures and Tables

**Table 1:** Ingredient list and proximate composition of experimental diets for rainbow trout (*O. mykiss*) juveniles, including *Scenedesmus* sp. (SCE).

Ingredient (%)	Diet CTRL	Diet SCE5
Fishmeal Super Prime	12.5	10.0
Fishmeal 60	8.0	6.6
Fish protein concentrate (CPSP 90)	2.5	2.5
Soy protein concentrate	12.5	12.5
Wheat gluten	9.0	9.0
Corn gluten	5.0	5.0
Soybean meal 48	15.0	15.0
Rapeseed meal	7.5	7.5
Wheat meal	7.0	7.0
Pea starch	4.0	4.0
Fish oil	14.5	13.4
Vitamin and mineral premix	1.0	1.0
Soy lecithin	1.0	1.0
Antioxidant	0.2	0.2
Sodium propionate	0.1	0.1
Guar gum	0.2	0.2
*Scenedesmus* sp.	-	5.0
**Proximate Composition**		
Protein (%)	48.1 ± 0.5	48.2 ± 0.3
Lipid (%)	18.2 ± 0.3	18.1 ± 0.4
Ash (%)	12.2 ± 0.2	12.1 ± 0.3
Gross energy (MJ kg^−1^) *	1.86 ± 0.3	1.86 ± 0.2

* Gross energy content was estimated by using the following: total carbohydrate × 17.2 J kg^−1^, fat × 39.5 J kg^−1^, and protein × 23.5 J kg^−1^.

**Table 2:** Fatty acid composition (% total fatty acids) of experimental diets for rainbow trout (*O. mykiss*) juveniles, including *Scenedesmus* sp.

Fatty Acid	Diet CTRL	Diet SCE-5
14:0	8.5 ± 0.2	8.2 ± 0.1
16:0	15.6 ± 0.3	15.2 ± 0.5
18:0	2.0 ± 0.4	1.9 ± 0.2
Total saturated	26.3 ± 1.1	25.3 ± 0.7
16:1 n-7	7.8 ± 0.2	7.8 ± 0.1
18:1 n-7	2.2 ± 0.1	2.2 ± 0.2
18:1 n-9	11.2 ± 0.3	11.4 ± 0.1
20:1 n-9	3.5 ± 0.2	3.6 ± 0.3
Total monounsaturated	28.1 ± 0.6	28.2 ± 0.8
18:2 n-6	3.7 ± 0.2	3.6 ± 0.2
18:3 n-6	0.1 ± 0.0	0.1 ± 0.0
20:3 n-6	0.2 ± 0.1	0.2 ± 0.1
20:4 n-6	1.1 ± 0.1	1.1 ± 0.3
22:4 n-6	0.1 ± 0.1	0.1 ± 0.1
22:5 n-6	0.3 ± 0.1	0.3 ± 0.1
Total n-6 PUFA	6.1 ± 0.4	6.0 ± 0.3
18:3 n-3	1.5 ± 0.1	1.4 ± 0.1
18:4 n-3	4.3 ± 0.2	4.3 ± 0.1
20:3 n-3	0.1 ± 0.1	0.1 ± 0.1
20:4 n-3	0.6 ± 0.1	0.7 ± 0.1
20:5 n-3	8.6 ± 0.3	8.7 ± 0.4
21:5 n-3	0.3 ± 0.1	0.3 ± 0.1
22:5 n-3	0.7 ± 0.1	0.7 ± 0.1
22:6 n-3	6.0 ± 0.1	6.5 ± 0.1
Total n-3 PUFA	24.1 ± 0.4	24.7 ± 0.3
Total PUFA	30.4 ± 0.6	30.8 ± 0.3

**Table 3:** Growth performance, body condition indexes and feed efficiency parameters in rainbow trout (*O. mykiss*) juveniles fed diets containing 5% of *Scenedesmus* sp. for 45 days.

Key Performance Indicator	Diets	
	CTRL	SCE-5
BW (g)	156.9 ± 3.0	154.2 ± 4.0
SL (cm)	20.0 ± 0.1	20.1 ± 0.4
SGR (% day^−1^)	1.63 ± 0.3	1.59 ± 0.4
K	1.9 ± 0.04	1.9 ± 0.10
HSI (%)	1.22 ± 0.08	1.24 ± 0.04
PVI (%)	6.14 ± 0.64	5.73 ± 0.66
FCR	1.15 ± 0.08	1.14 ± 0.06
FI (%)	1.80 ± 0.04	1.81 ± 0.07

**Table 4:** Fillet color analysis from rainbow trout (*O. mykiss*) juveniles fed diets containing 5% of *Scenedesmus* sp. for 45 days. Different letters denote the presence of statistically significant differences between both groups (*t*-test; *p* < 0.05).

Parameter	Diets	
	CTRL	SCE-5
L*	38.9 ± 0.3	39.0 ± 1.0
a*	4.0 ± 0.4	4.8 ± 0.2
b*	4.6 ± 0.1 b	17.5 ± 0.8 a
C*	6.7 ± 0.2 b	18.0 ± 0.7 a
h*	43.5 ± 1.4 b	76.8 ± 1.8 a

**Table 5:** Proximate composition of the fillet and liver of rainbow trout (*O. mykiss*) juveniles fed diets containing 5% of *Scenedesmus* sp. for 45 days. Different letters denote significant differences among diets (*t*-test, *p* < 0.05).

Parameter	Fillet		Liver	
	Diet CTRL	Diet SCE-5	Diet CTRL	Diet SCE-5
Protein (%)	86.8 ± 4.3	84.6 ± 3.3	62.6 ± 4.5	64.3 ± 3.4
Lipid (%)	6.7 ± 1.3	7.3 ± 0.7	19.2 ± 0.5 a	17.6 ± 1.3 b
Carbohydrates (%)	2.6 ± 0.4	2.2 ± 0.4	17.2 ± 1.3	17.1 ± 1.5
Ash (%)	3.8 ± 0.9	4.9 ± 1.1	0.9 ± 0.1	1.0 ± 0.1

**Table 6:** Fillet and liver fatty acid composition (% total fatty acids) in rainbow trout (*O. mykiss*) juveniles fed diets containing 5% of *Scenedesmus* sp. for 45 days. Different letters denote significant differences among diets (*t*-test, *p* < 0.05). Abbreviation: nd, non-detected.

Fatty Acid	Fillet		Liver	
	Diet CTRL	Diet SCE-5	Diet CTRL	Diet SCE-5
14:0	1.7 ± 0.2	1.8 ± 0.1	1.5 ± 0.1	1.4 ± 0.1
15:0	0.2 ± 0.1	0.3 ± 0.1	0.2 ± 0.1	0.2 ± 0.1
16:0	16.6 ± 0.5	17.0 ± 0.3	15.4 ± 1.4	15.3 ± 1.1
18:0	2.5 ± 0.4	3.5 ± 1.1	3.0 ± 0.2	3.5 ± 0.3
Total saturated	22.4 ± 2.1	22.6 ± 0.9	20.2 ± 1.1	20.4 ± 0.7
16:1n-7	4.8 ± 0.1	4.7 ± 0.1	4.6 ± 0.3	3.9 ± 0.3
18:1n-7	3.5 ± 0.2	3.6 ± 0.1	nd	nd
18:1n-9	16.2 ± 0.1	15.8 ± 0.1	15.7 ± 0.6	16.1 ± 0.5
20:1n-9	5.8 ± 0.2	5.4 ± 0.3	4.9 ± 0.5	4.7 ± 0.4
Total monounsaturated	33.1 ± 0.7 a	29.5 ± 1.4 b	29.0 ± 0.9	28.1 ± 1.1
18:2n-6	6.7 ± 0.2	6.5 ± 0.2	4.5 ± 0.1	4.7 ± 0.2
18:3n-6	0.1 ± 0.1	0.1 ± 0.0	0.1 ± 0.0	0.2 ± 0.1
20:3n-6	0.3 ± 0.1	0.5 ± 0.2	0.4 ± 0.1	0.4 ± 0.1
20:4n-6	1.9 ± 0.2	2.1 ± 0.3	2.1 ± 0.2	2.0 ± 0.2
22:4n-6	0.1 ± 0.1	0.2 ± 0.1	nd	nd
22:5n-6	0.3 ± 0.1	0.3 ± 0.1	0.3 ± 0.1	0.3 ± 0.1
Total n-6 PUFA	9.5 ± 0.4	9.7 ± 0.3	7.5 ± 0.4	7.4 ± 0.3
18:3n-3	1.5 ± 0.1 a	1.1 ± 0.2 b	1.0 ± 0.2	1.1 ± 0.2
18:4n-3	1.6 ± 0.2	1.3 ± 0.3	0.6 ± 0.1	0.7 ± 0.2
20:3n-3	0.1 ± 0.1	0.2 ± 0.1	0.1 ± 0.1	0.1 ± 0.0
20:4n-3	0.9 ± 0.1	1.1 ± 0.1	0.5 ± 0.3	0.5 ± 0.2
20:5n-3	8.3 ± 0.3	8.1 ± 0.4	7.3 ± 0.5	6.6 ± 0.6
21:5n-3	0.2 ± 0.1	0.3 ± 0.1	0.1 ± 0.1	0.1 ± 0.0
22:5n-3	1.2 ± 0.1 b	1.4 ± 0.1 a	1.6 ± 0.3	1.4 ± 0.2
22:6n-3	30.3 ± 0.1 b	32.5 ± 0.3 a	32.3 ± 0.8	33.7 ± 1.1
Total n-3 PUFA	44.1 ± 1.2 b	46.0 ± 1.0 a	43.5 ± 1.2	44.2 ± 1.5
Total PUFA	53.6 ± 1.0	55.7 ± 1.1	50.9 ± 1.0	51.6 ± 2.3

**Table 7:** Lipid classes composition (in %) in the liver of rainbow trout (*O. mykiss*) juveniles fed diets containing 5% of *Scenedesmus* sp. for 45 days. Different letters denote significant differences among diets (*t*-test, *p* < 0.05).

Lipid	Diet CTRL	Diet SCE-5
SM.	1.0 ± 0.1	1.4 ± 0.2
LysoPC	2.7 ± 0.4	3.7 ± 0.8
PC	24.1 ± 1.8 b	29.6 ± 3.4 a
PS + Pi	3.8 ± 1.3 b	8.0 ± 0.6 a
LysoPE	1.4 ± 0.1 b	2.4 ± 0.1 a
PE	12.5 ± 0.9	12.9 ± 0.8
Total phospholipids	46.1 ± 1.9 b	58.0 ± 2.6 a
CHOL	15.2 ± 1.6 a	10.1 ± 0.9 b
FFA	18.6 ± 1.8 a	13.9 ± 1.1 b
TAG	9.1 ± 3.1	8.2 ± 4.1
SE + W	10.6 ± 0.7	9.8 ± 1.0
Total neutral lipids	53.6 ± 1.8 a	42.0 ± 5.6 b

Abbreviations: SM, sphingomyelin; LysoPC, lysophosphatydylcholine; PC phosphatydylcholine; PS + Pi, phosphatidylserine and phosphatidylinositol; LysoPE, lysophosphatidylethanolamine; PE, phosphatidylethanolamine; total PL, total phospholipids; CHO, cholesterol; FFA, free fatty acids; TAG, triacylglycerides; SE + W, sterols and waxes; total NL, total neutral lipids.

**Table 8:** Lipid peroxidation levels and activity of oxidative stress enzymes in the fillet and liver of rainbow trout (*O. mykiss*) juveniles fed diets containing 5% of *Scenedesmus* sp. for 45 days. The asterisk denotes differences in enzyme activity levels between the fillet and liver regardless of the diet considered (ANOVA, *p* < 0.05).

Parameter	Fillet		Liver	
	Diet CTRL	Diet SCE-5	Diet CTRL	Diet SCE-5
LPO (mmol MDA 100 g^−1^)	109.7 ± 1.0	110.7 ± 5.2	112.9 ± 9.1	116.5 ± 6.8
SOD (% enzyme inhibition)	32.3 ± 9.6	33.9 ± 3.3	67.8 ± 5.6 *	65.6 ± 2.7 *
CAT (mmol min^−1^ mg protein^−1^)	158.8 ± 43.2 *	171.7 ± 31.5 *	63.0 ± 26.8	33.0 ± 6.5
GST (mmol min^−1^ mg protein^−1^)	1315.6 ± 157.9 *	1505.8 ± 203.4 *	32.9 ± 7.6	33.5 ± 10.3
GPX (mmol min^−1^ mg protein^−1^)	793.7 ± 110.5 *	732.2 ± 187.3 *	28.3 ± 4.7	23.5 ± 4.9
GR (mmol min^−1^ mg protein^−1^)	641.5 ± 143.0 *	600.8 ± 141.3 *	2.3 ± 0.5	2.4 ± 0.2

Abbreviations: CAT, catalase; LPO, lipid peroxidation; GPX, glutathione peroxidase; GR, glutathione reductase; GST, glutathione S-transferase; SOD, superoxide dismutase.

**Table 9:** Specific activity of pancreatic, gastric and brush border intestinal enzymes from rainbow trout (*O. mykiss*) juveniles fed diets containing 5% of *Scenedesmus* sp. for 45 days. Different letters denote significant differences among diets (*t*-test; *p* < 0.05).

Enzyme Activity (U mg Protein^−1^)	Diet CTRL	Diet SCE-5
Stomach/pyloric caeca		
Total alkaline proteases	0.312 ± 0.009 b	0.391 ± 0.006 a
Trypsin	0.257 ± 0.008 b	0.2931 ± 0.006 a
Chymotrypsin	0.111 ± 0.007 b	0.132 ± 0.003 a
α-amylase	1.11 ± 0.09	1.19 ± 0.11
Bile salt-activated lipase	1.21 ± 0.11	1.31 ± 0.07
Pepsin	5.21 ± 0.75	5.45 ± 0.32
Intestine		
Alkaline phosphatase	0.851 ± 0.011	0.798 ± 0.009
Aminopeptidase N	0.051 ± 0.003	0.060 ± 0.010
Maltase	12.1 ± 1.25	13.4 ± 2.11

**Table 10:** Villi length and goblet cell density from the anterior and posterior intestine of rainbow trout (*O. mykiss*) juveniles fed diets containing 5% of *Scenedesmus* sp. for 45 days.

Parameter	Diet CTRL	Diet SCE-5
Anterior intestine		
Villi length (µm)	650.5 ± 123.5	638.9 ± 79.6
Goblet cell density *	2.5 ± 0.2 b	3.5 ± 0.2 a
Posterior intestine		
Villi length (µm)	421.2 ± 78.3	433.3 ± 36.9
Goblet cell density *	1.5 ± 0.3	1.4 ± 0.2

* Goblet cell counts in intestinal villi were expressed over a contour length of 100 μm.

**Table 11:** Hematocrit, humoral immune parameters, and peroxidase and myeloperoxidase levels in serum of trout (*O. mykiss*) juveniles fed diets containing 5% of *Scenedesmus* sp. for 45 days.

Parameter	Diet CTRL	Diet SCE-5
Htc (%)	40.0 ± 1.8	42.6 ± 1.4
Protease (U mg protein^−1^)	0.51 ± 0.08	0.70 ± 0.21
Lysozyme (KU mL^−1^)	1287.9 ± 130.4	1235.3 ± 164.7
ACP (ACH_50_ mL^−1^)	122.6 ± 10.0	126.8 ± 2.2
Bacteriolytic activity (% Abs)	3.6 ± 0.8	4.7 ± 2.1
Peroxidase (mU mL^−1^)	0.257 ± 0.092	0.505 ± 0.090
MPO (µU mL^−1^)	0.054 ± 0.016	0.050 ± 0.020

Abbreviations: ACP, activity of the complement; Htc, hematocrit; MPO, myeloperoxidase level.

## Supplementary Materials

The following are available online at https://www.mdpi.com/2076-2615/10/9/1656/s1, Figure S1: Examples of fillet and serum color from *Oncorhynchus mykiss* juveniles fed diets containing 5% of *Scenedesmus* sp. for 45 days, Figure S2: Histological organization of the liver (hepatic parenchyma) and anterior-mid intestine of *Oncorhynchus mykiss* juveniles fed diets containing 5% of *Scenedesmus* sp. for 45 days. Three images from different specimens are shown per dietary group in order to show individual variation. Staining: hematoxylin-eosin.
