# Supplementary material for: The Inclusion of the Microalga Scenedesmus sp. in Diets for Rainbow Trout, Onchorhynchus mykiss, Juveniles
Source: Animals (Basel). 2020 Sep 15;10(9):1656. doi: 10.3390/ani10091656 (PMC7552274; doi:10.3390/ani10091656)
Supplement: Supplementary file 1 [file animals-10-01656-s001.pdf]

Article

The Inclusion of the Microalga *Scenedesmus* sp. in Diets for Rainbow Trout, *Onchorhynchus mykiss*, Juveniles

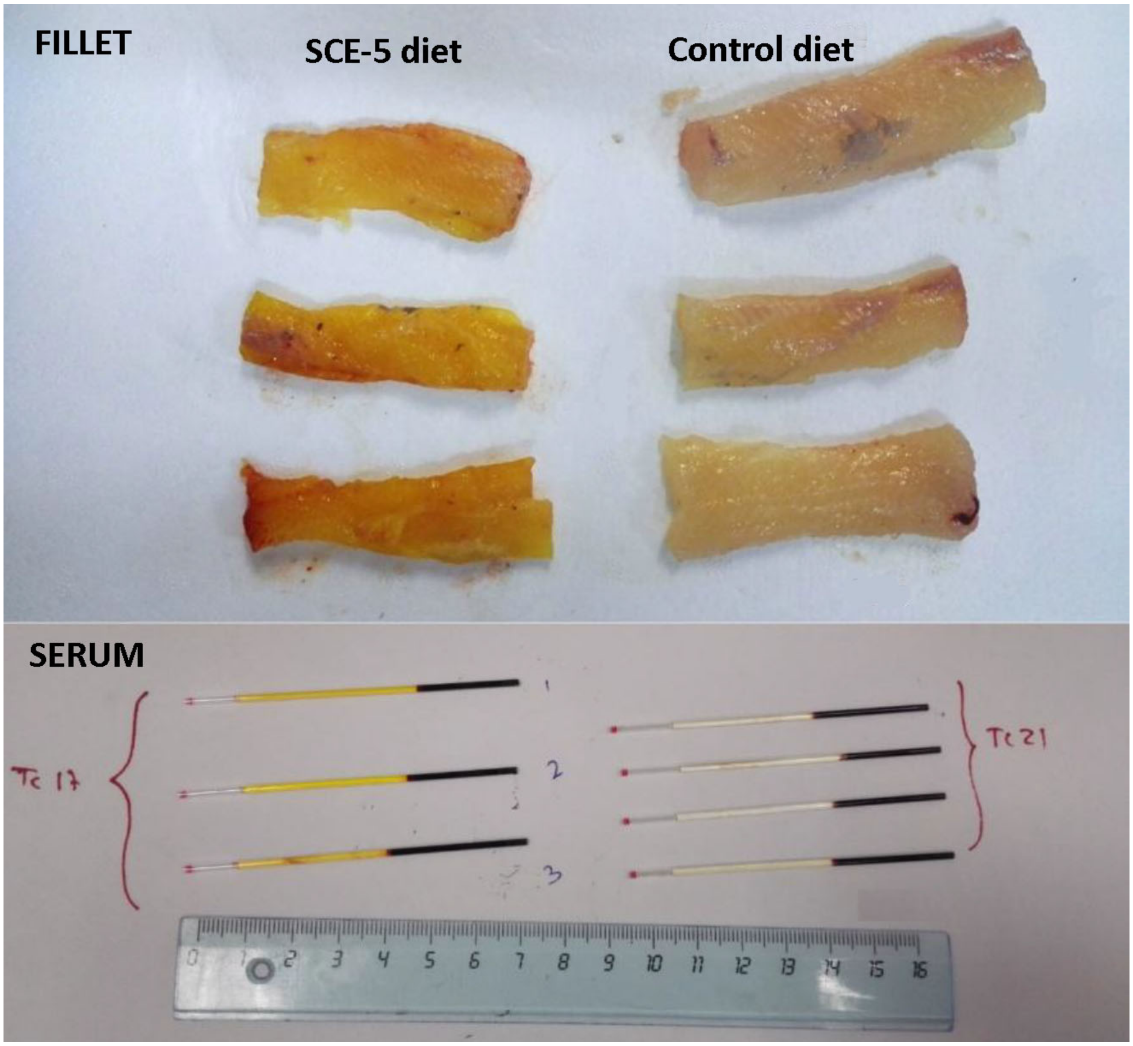

Figure S1. Examples of fillet and serum color from *Oncorhynchus mykiss* juveniles fed diets containing 5% of *Scenedesmus* sp. for 45 days.

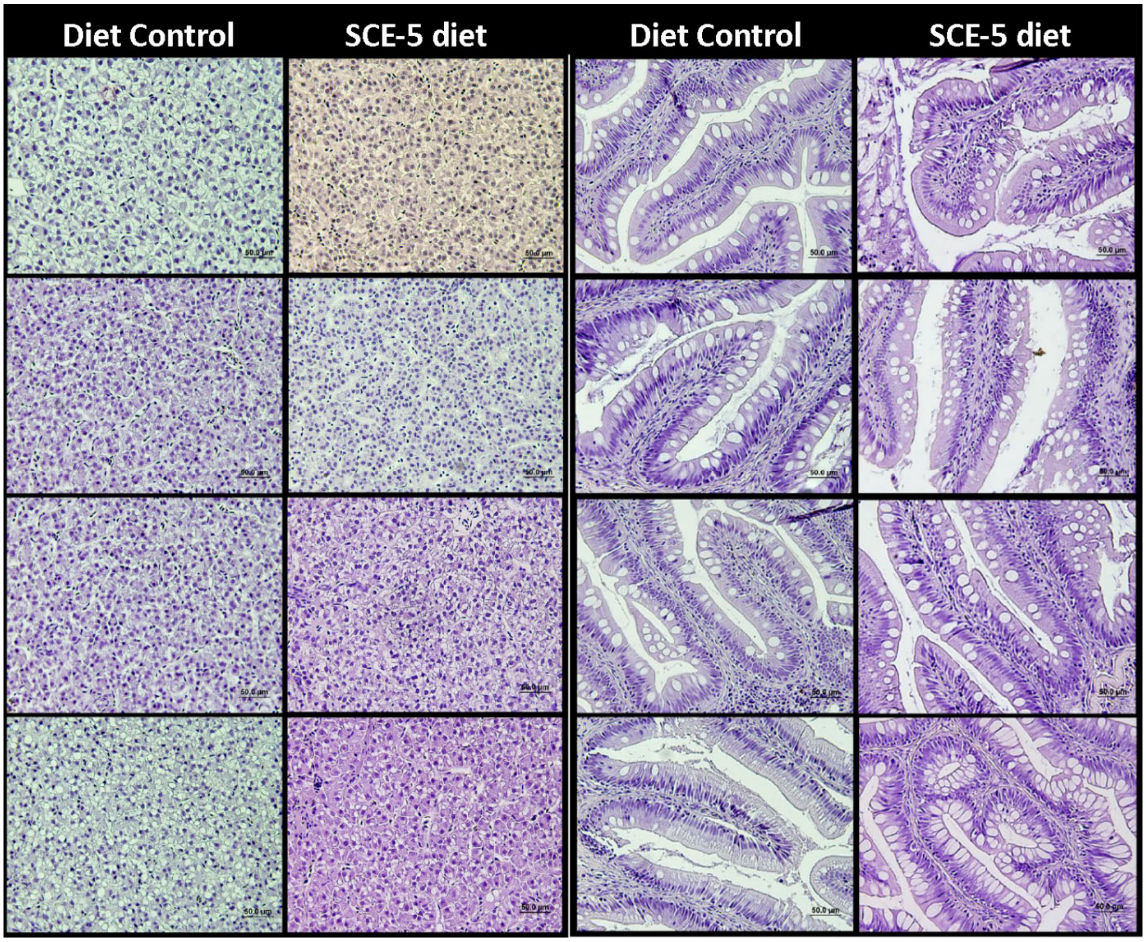

Figure S2. Histological organization of the liver (hepatic parenchyma) and anterior-mid intestine of *Oncorhynchus mykiss* juveniles fed diets containing 5% of *Scenedesmus* sp. for 45 days. Three images from different specimens are shown per dietary group in order to show individual variation. Staining: hematoxylin-eosin.
